# Referral Patterns of Community Health Workers Diagnosing and Treating Malaria: Cluster-Randomized Trials in Two Areas of High- and Low-Malaria Transmission in Southwestern Uganda

**DOI:** 10.4269/ajtmh.16-0598

**Published:** 2016-12-07

**Authors:** Sham Lal, Richard Ndyomugenyi, Pascal Magnussen, Kristian S. Hansen, Neal D. Alexander, Lucy Paintain, Daniel Chandramohan, Siân E. Clarke

**Affiliations:** 1 Department of Disease Control, Faculty of Infectious Tropical Diseases, London School of Hygiene and Tropical Medicine, London, United Kingdom; 2 Vector Control Division, Ministry of Health, Kampala, Uganda; 3 Department of International Health, Immunology and Microbiology, Faculty of Health and Medical Sciences, University of Copenhagen, Copenhagen, Denmark; 4 Department of Veterinary Disease Biology, Faculty of Health and Medical Sciences, University of Copenhagen, Copenhagen, Denmark; 5 Medical Research Council Tropical Epidemiology Group, Department of Infectious Disease Epidemiology, Faculty of Epidemiology and Population Health, London School of Hygiene and Tropical Medicine, London, United Kingdom; 6 Institute of Public Health, University of Copenhagen, Copenhagen, Denmark

## Abstract

Malaria-endemic countries have implemented community health worker (CHW) programs to provide malaria diagnosis and treatment to populations living beyond the reach of health systems. However, there is limited evidence describing the referral practices of CHWs. We examined the impact of malaria rapid diagnostic tests (mRDTs) on CHW referral in two cluster-randomized trials, one conducted in a moderate-to-high malaria transmission setting and one in a low-transmission setting in Uganda, between January 2010 and July 2012. All CHWs were trained to prescribe artemisinin-based combination therapy (ACT) for malaria and recognize signs and symptoms for referral to health centers. CHWs in the control arm used a presumptive diagnosis for malaria based on clinical symptoms, whereas intervention arm CHWs used mRDTs. CHWs recorded ACT prescriptions, mRDT results, and referral in patient registers. An intention-to-treat analysis was undertaken using multivariable logistic regression. Referral was more frequent in the intervention arm versus the control arm (moderate-to-high transmission, *P* < 0.001; low transmission, *P* < 0.001). Despite this increase, referral advice was not always given when ACTs or prereferral rectal artesunate were prescribed: 14% prescribed rectal artesunate in the moderate-to-high setting were not referred. In addition, CHWs considered factors alongside mRDTs when referring. Child visits during the weekends or the rainy season were less likely to be referred, whereas visits to CHWs more distant from health centers were more likely to be referred (low transmission only). CHWs using mRDTs and ACTs increased referral compared with CHWs using a presumptive diagnosis. To address these concerns, referral training should be emphasized in CHW programs as they are scaled-up.

## Introduction

Early diagnosis and effective treatment of malaria is an essential strategy to reduce the high under-5 child morbidity and mortality associated with malaria in sub-Saharan Africa (SSA).^1^,^2^ To address the disease burden, United Nations Children's Fund, World Health Organization (WHO), and Save the Children have supported SSA countries to implement national community-based programs such as integrated community case management (iCCM) for the diagnosis and treatment of malaria, pneumonia, and diarrhea.^3^,^4^ The programs typically train laypersons with limited medical education, as community health workers (CHWs) that are able to provide case management services in communities with poor access to public health facilities.

Early community studies found CHWs were able to reduce malaria morbidity and mortality using a presumptive diagnosis based on clinical symptoms.^5^,^6^ However, WHO now recommends all suspected cases of malaria should be tested with a malaria rapid diagnostic test (mRDT) and on confirmation, treated with an artemisinin-based combination therapy (ACT).^7^ Following WHO recommendations, and supported by strong evidence that CHWs can effectively treat uncomplicated malaria and adhere to mRDT results,^8^,^9^ many SSA countries have begun to introduce mRDTs and ACTs at the community level. Community programs usually also train CHWs to refer children who present with complicated illnesses to a health center for further management. A referral pathway is an important element of primary health care that ensures the continuity of a child's care from the community to health center. It helps to ensure that professional health-care workers manage complex illnesses in medically equipped health facilities, whereas CHWs manage the uncomplicated illnesses in the community. Failure to refer or comply with referral advice may increase the risk of medical complications. Despite the large body of evidence on implementing and scaling-up national CHW programs, currently there is relatively little evidence on referral practices by CHWs using both mRDTs and ACTs. Two small-scale studies in Tanzania and Sierra Leone found CHWs referred between 5% and 15% of children and very few children completed referral.^10^,^11^ Filling the evidence gap on referral was identified as important component to improve the effectiveness, implementation, and scale-up of community-based programs by the international taskforce on iCCM.^12^

We undertook an exploratory examination of referrals made by CHWs as part of two large trials that evaluated the impact of mRDTs on appropriately targeted ACTs: one trial was conducted in a moderate-to-high malaria transmission setting and the other in a low-transmission setting.^13^ The trials provided an opportunity to yield insights on how referral patterns can change following the introduction of mRDTs and the objective of this referral study was to examine the referrals made by CHWs and explore the factors associated with referral when CHWs diagnosed malaria using either a presumptive or mRDT-based diagnosis.

## Methods

### Study area and participants.

Both trials were conducted in Rukungiri District, Western Region, Uganda, one in a moderate-to-high malaria transmission setting in Bwambara subcounty (980–1,200 m above sea level), the other in a low-transmission setting in Nyakishenyi subcounty (1,064–2,157 m above sea level). The 2002 census for Uganda reported a population of 28,900 in the moderate-to-high transmission setting and 32,000 in the low-transmission setting, with more than 85% living in rural areas in both settings.^14^ The main occupation in both settings was subsistence agriculture and the majority of the population belonged to either Bahororo or Bakiga ethnic groups. The climate in the area is characteristic of east African tropics with mean annual temperatures between 16 and 25°C and a pronounced bimodal pattern of annual rainfall with a long rainy season between September and December and a short rainy season from March to May. Malaria transmission is perennial with peaks in incidence shortly after the rains.

The public health system in each transmission setting comprises three health centers: two are classed as public health center IIs (HCII), which only provide outpatient and community outreach services, and the third is a health center III (HCIII) that provides basic preventive and curative care and supervises lower level HCIIs. HCIIIs also have services for diagnosis, maternity care and act as the first referral cover for the subcounty.^15^

### Trial design.

**Figure 1.**
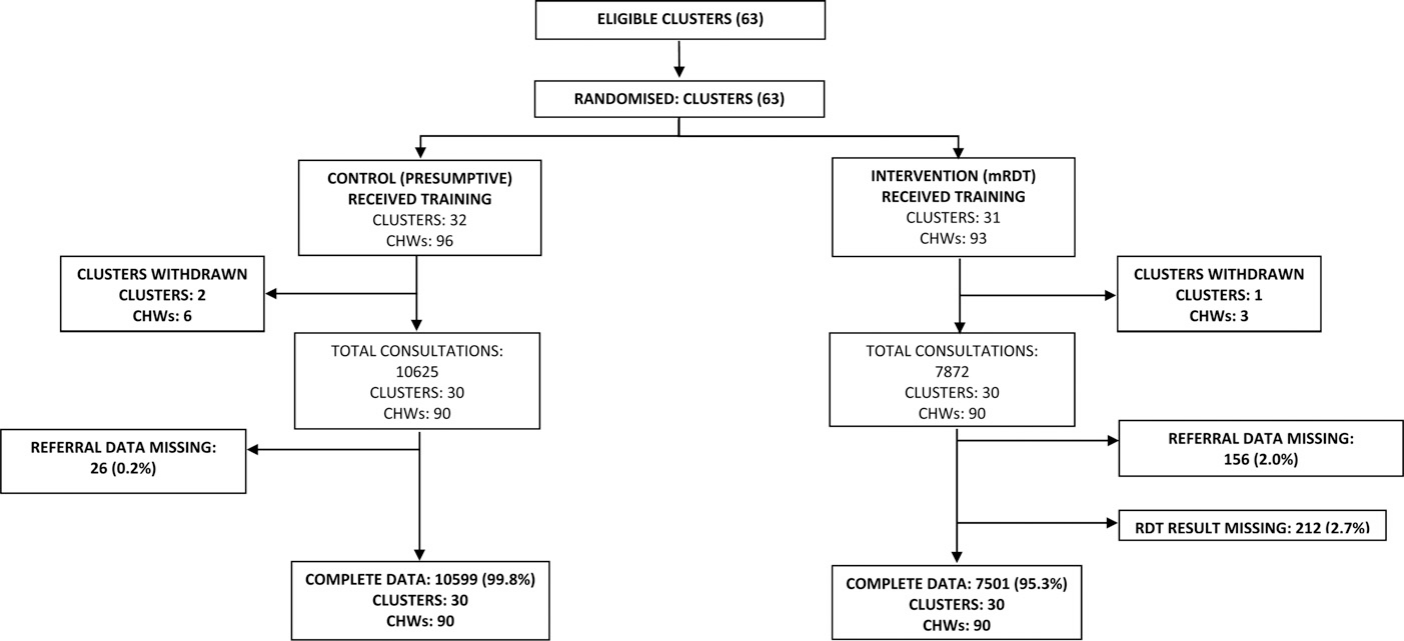
Trial profile for the moderate–to–high transmission setting.

**Figure 2.**
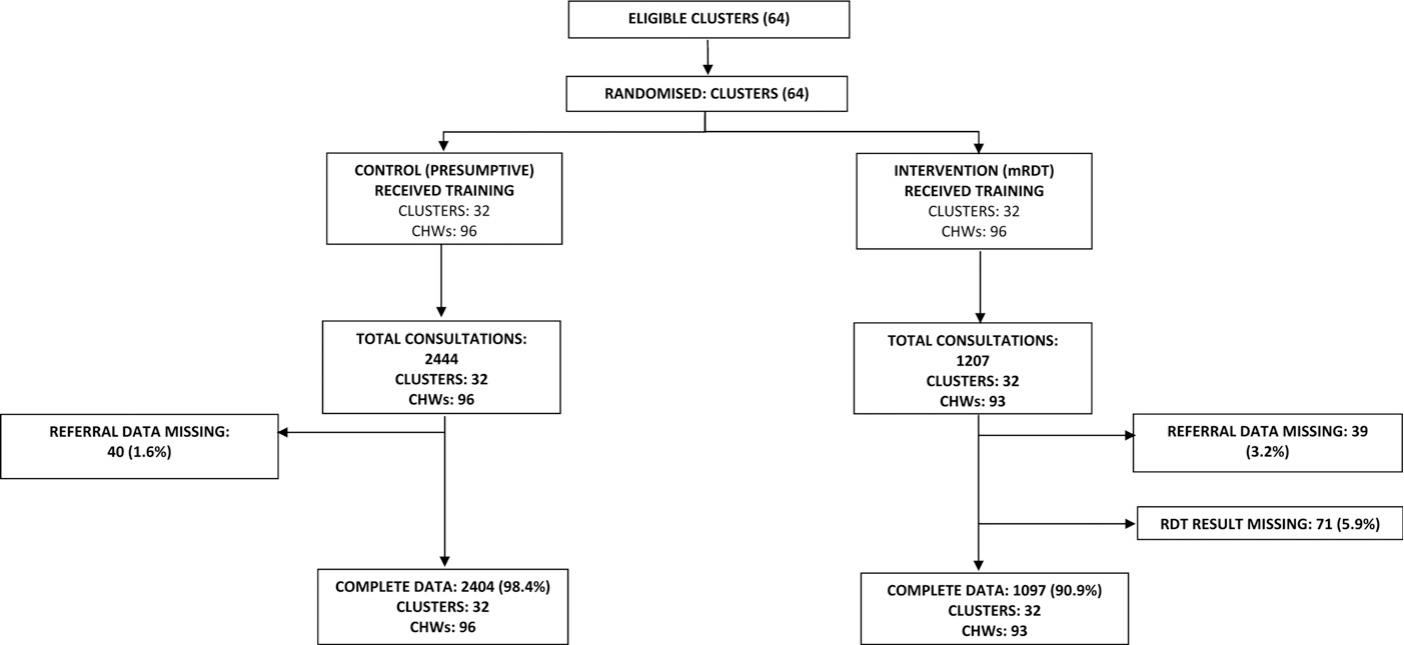
Trial profile for the low-transmission setting.

A two-arm cluster-randomized controlled trial design was used to evaluate the primary outcome of the main trials, to compare the effectiveness of CHWs using mRDTs versus CHWs using a presumptive diagnosis to treat malaria with an ACT. In each transmission setting, a series of community meetings were held to explain the purpose of the research and to elect three adults per village for CHW training. All elected CHWs provided written and informed consent to take part in the trial and in each setting villages (clusters) were randomized in 1:1 ratio to either the intervention arm (mRDT diagnosis) or control arm (presumptive diagnosis) using Epi-Info (Centers for Disease Control and Prevention, Atlanta, GA). In the moderate-to-high transmission setting, 63 villages were randomized (Figure 1

), and in the low-transmission setting, 64 were randomized (Figure 2

). In addition, shortly before the trials began, community sensitization was carried out in both transmission settings. Key messages included that not all fevers are malaria and a diagnostic test is advisable before treatment with an ACT, a quick malaria test (mRDT) could test for malaria, and these tests were available from CHWs in the intervention arm.

### CHW intervention.

**Figure 3.**
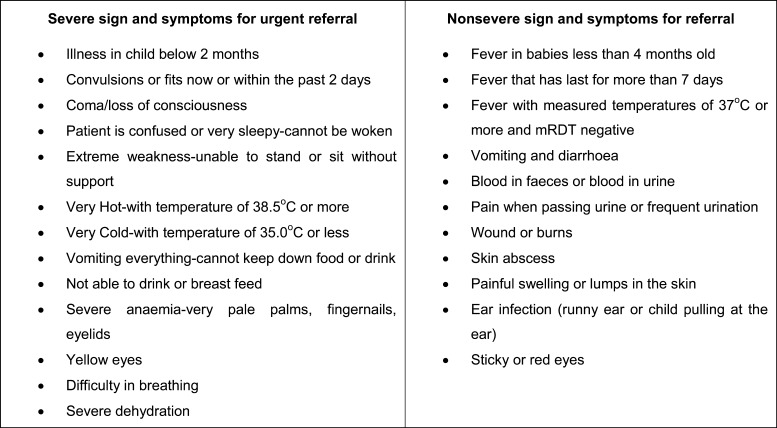
Severe and non-severe signs and symptoms community health workers (CHWs) were trained to identify at the time of a child visit.

In January 2010, 381 CHWs received training (192 and 189 in the moderate-to-high- and low-transmission settings, respectively) in the management of febrile children during 3- to 4-day workshops. The training was based on a manual with simplified pictorial treatment algorithms (job aids) to help with malaria diagnosis (Supplemental Figure 1). The key topics included how to take a basic clinical history, physical examination skills, and counseling caregivers. All CHWs were also trained on how to identify nonsevere and severe signs and symptoms, which would require immediate referral to the nearest health center for further management. Severe signs and symptoms included convulsions or fits, extreme weakness, coma, loss of consciousness, and very hot body temperature of 38.5°C or more, whereas nonsevere signs and symptoms included wounds or burns, ear infections, sticky or red eyes, and vomiting and diarrhea (Figure 3). Members of the District Health Office, research staff, and health center workers conducted the workshops. These were interactive and included presentations, role-plays, demonstrations, and supervised clinical practice at health centers.

CHWs in the intervention arm were trained to diagnose uncomplicated malaria with an mRDT (First Response^®^ MalariaHRP2, Premier Medical Corp, Nani Daman, India) and prescribe test-positive children with an age-dependent dose of ACT (artemether–lumefantrine, Lumartem^®^, Mumbai, India). CHWs were also trained to prescribe prereferral treatment, rectal artesunate to children who visited with signs and symptoms of severe malaria, and to refer them immediately to the nearest health center. CHWs were trained not to prescribe ACT to children who were mRDT negative, and to refer those who had signs and symptoms of severe or nonsevere illnesses (Supplemental Figure 1). In contrast, CHWs in the control arm were trained to make a presumptive diagnosis based on the clinical symptoms of malaria if a child had a fever or history of fever without any other obvious causes of fever and prescribe an ACT; if there were severe signs and symptoms of disease, they were trained to prescribe rectal artesunate and refer them immediately to the nearest health center (Supplemental Figure 1).

CHWs were also trained on how to manage stocks of consumables such as ACTs, gloves, cotton wool, and mRDTs (intervention arm only). They recorded basic demographic details about the child, such as age, sex, their village of residence, and the head of household's name on treatment recording forms. CHWs also reported the clinical history of the febrile illness such as the child's temperature, how long ago the fever started, and whether the child slept under a net the previous night. Finally, CHWs recorded whether an ACT or rectal artesunate was prescribed, the test result (intervention arm only), and whether they referred the child. In the event of a referral, CHWs were asked to classify it as a severe or nonsevere referral and subsequently complete a referral form and mark the signs and symptoms they identified. The research team collected treatment recording forms and referral forms during monthly meetings (further details of the trial and training materials are available at www.actconsortium.org/RDThomemanagement).

### Data collection.

The trials in the moderate-to-high- and low-transmission settings started in May 2010 and June 2010, respectively. Between May/June 2010 and December 2010, CHWs were visited by field coordinators at least once per week to discuss concerns or difficulties in carrying out their roles and collected CHWs treatment recording forms, referral forms, and stock cards for ACTs. From January 2011 to the end of the trial in July 2012, this supervision of CHWs was scaled back and limited to monthly meetings to reflect an operational program.

To examine the patterns of referrals CHW made, we collected data from the treatment recording and referral forms for the 19-month “operational” intervention period (January 2011 to July 2012). Previous studies suggested a number of characteristics that may influence referral patterns, these included the child's age, sex, net use the previous night, and the duration of fever.^16^ In addition, the day of consultation (weekday/weekend) was derived from the date of the child's visit and a rainy season variable was defined to coincide with the rains that occurred in the months September–December and March–May. Finally, global positioning system coordinates were taken in the center of each village and at the health center to measure Euclidean (straight line) distance from the center of a village to the nearest health center.

### Statistical methods.

All data were double entered and verified using Microsoft Access 2007 (Microsoft Inc., Redmond, WA). Village distances to the nearest health center were calculated using ArcGIS Desktop 10.3 (ESRI, CA). All data were analyzed using STATA version 14.1 (STATA Corporation, College Station, TX).

The primary outcome for this analysis was the proportion of children that were referred by CHWs and a different method of analysis was undertaken for each trial. In the moderate-to-high transmission setting, three clusters (two control arms and one intervention arm) withdrew from the trial after randomization and contributed no data to the analyses, therefore, a modified intention to treat analysis was undertaken with 30 clusters in each arm (Figure 1). In the low-transmission setting, no clusters withdrew after randomization and all clusters contributed data to the primary outcome analysis, allowing an intention-to-treat analysis (Figure 2).

We present child and cluster characteristics of the study population in each arm using descriptive statistics (proportions, means). We also assessed whether there were important differences between the arms by examining the size of any child- or cluster-level imbalances between the study arms after randomization. Unadjusted and adjusted analyses were undertaken for each trial and clustering of data at the village level was accounted for using logistic regression models with random effects.^17^ To understand the etiology of referral and identify a set of independent factors associated with referral, all factors identified a priori were included in multivariable models. In both the univariable and multivariable models, the significance of factors was compared using a likelihood ratio test.

### Ethics statement.

The main trial was approved by the Uganda National Council for Science and Technology (Ref no. HS 555) and London School of Hygiene and Tropical Medicine Ethics Committee (Ref no. 5595). Before randomization, meetings were held with community leaders in each village to explain the study objectives and procedures. Written informed consent was obtained from village leaders and CHWs to participate in the trial. At the time of visit with a CHW, children refusing an mRDT received presumptive treatment. The study was registered with ClinicalTrials.gov. Identifier NCT01048801 on January 13, 2010. An independent data safety and monitoring board reviewed the protocol and the analytical plan.

## Results

### Study population.

During the 19-month intervention period (January 2011 to July 2012) 18,100 child visits were reported by CHWs in 60 villages in the moderate-to-high transmission setting (7,501 in the control arm and 10,599 in the intervention arm) (Figure 1

). In the low-transmission setting, CHWs saw 3,501 children in 64 villages; 2,404 and 1,097 in the control and intervention arms, respectively (Figure 2). There were similarities in both transmission settings and trial arms: the majority of children were aged between 1 and 5 years, approximately half were female, more than 86% had slept under a net the previous night, and nearly all (> 82.0%) resided in the same village as the CHW (Table 1). We also examined characteristics that were different between the arms after randomization and found a larger proportion of children visited control arm villages within 24 hours after the onset of fever symptoms, compared with the intervention arm (moderate-to-high transmission setting, 94.4% versus 85.1%; low-transmission setting, 88.3% versus 78.4%; control and intervention arms, respectively). Similarly, a greater proportion of visits occurred during the rainy season in the intervention arm villages compared with the control arm (moderate-to-high transmission setting, 63.2% versus 56.3%; low-transmission setting, 65.5% versus 59.7%). The notable difference between the transmission settings was that children in the low-transmission setting lived more than 5 km away from the nearest public health center compared with children in the moderate-to-high transmission setting (Table 1).

### Referral and treatment practices.

CHWs in the low-transmission setting referred a greater proportion of child visits than CHWs in the moderate-to-high transmission setting (31.3% [1,096/3,501] versus 15.2% [2,760/18,100]). Within each transmission setting, CHWs referred more frequently in the intervention arm compared with the control arm (moderate-to-high transmission setting, 35.3% versus 1.0%, *P* < 0.001; low-transmission setting, 71.3% versus 13.1%, *P* < 0.001; Tables 2 and 3). An examination of referrals according to mRDT results in the intervention arms found 61.7% (2,558/4,147) of mRDT-negative children were referred compared with 2.8% (93/3,355) of mRDT-positive children in the moderate-to-high transmission setting (Table 2). While in the low-transmission setting, 72.4% (770/1,064) of mRDT-negative children were referred compared with 38.2% (13/34) mRDT-positive children (Table 3).

CHWs ACT prescribing patterns and their referral practices are also shown in Tables 2 and 3. Referral was more frequent when an ACT was not prescribed compared with when an ACT was prescribed in both settings (moderate-to-high transmission setting, 61.1% versus 0.6%; low-transmission setting, 74.3% versus 9.0%). In both settings, the use of prereferral rectal artesunate was low with less than 2% of all visits receiving rectal artesunate (0.4% [70/18,100] and 1.7% [59/3,501] in moderate-to-high and low-transmission setting, respectively). Not all children receiving rectal artesunate were referred: in the moderate-to-high transmission setting, 14.3% (10/70) of children prescribed rectal artesunate were not referred; in contrast, in the low-transmission setting this figure was 1.7% (1/59).

CHWs classified referrals as nonsevere or severe depending on the signs and symptoms they identified (Tables 2 and 3). Children visiting with severe signs and symptoms were more common in the low-transmission setting compared with the moderate-to-high transmission setting (11.5% versus 5.5%). Nonsevere signs and symptoms for referrals were more likely to be referred in intervention arm compared with control arm (62.6% versus 24.7%, *P* < 0.001) in the moderate-to-high transmission setting. There was no difference between the trial arm and the type of referrals CHWs made in the low-transmission setting (*P* = 0.120).

### Referral signs and symptoms.

The specific signs and symptoms for referring a child were collected using a referral form separate to the treatment recording form, and Tables 4 and 5 list the signs and symptoms CHWs identified using this referral form. On average, CHWs recorded three or more signs and symptoms for referral on each referral form. However, it is important to note that unfortunately, despite the number of referrals made and recorded in the treatment recording form, CHWs returned very few referral forms to the research team. In the moderate-to-high transmission setting, only 260 (9.4%) referral forms were collected despite CHWs reporting 2,760 referrals on the treatment recording form, similarly in the low-transmission setting, only 204/1,096 (18.6%) forms were collected. The reason for this disparity is unknown, though it is possible that CHWs only informed patients verbally that additional treatment from a health facility was advisable.

In the intervention arm of each transmission setting, the most frequently reported nonsevere signs and symptoms related to referral were “fever and mRDT negative,” “vomiting/diarrhea,” and “pain when passing urine” (Table 4). In contrast, the most frequent severe signs and symptoms related to referral were “high fever,” “difficulty in breathing,” and the “inability to drink or breastfeed” (Table 5). When the signs and symptoms of referral were examined according to mRDT test result, there were generally very few signs and symptoms recorded for mRDT-positive referrals compared with mRDT-negative referrals, in both transmission settings (Supplemental Tables 1 and 2).

### Factors associated with referral.

The results of the logistic regression analyses to explore child factors associated with referral are shown in Tables 6 and 7 for the moderate-to-high and low-transmission settings, respectively. In the adjusted analysis of both transmission settings we found an independent relationship between ACT prescription and referral. Children who were prescribed ACTs were very unlikely to be referred when compared with children who were not prescribed ACTs (moderate-to-high transmission setting: odds ratio [OR] = 0.003; 95% confidence interval [CI] = 0.002–0.004, *P* < 0.001; low-transmission setting: OR = 0.03, 95% CI 0.01–0.05, *P* < 0.001). The adjusted analysis also showed CHWs in the intervention arm using mRDTs were more than twice as likely to refer visits compared with CHWs using a presumptive diagnosis; however, this finding was not statistically significant (moderate-to-high transmission setting OR = 2.07; 95% CI = 0.97–4.41; *P* = 0.06, low-transmission setting OR = 2.07; 95% CI = 0.90–4.79; *P* = 0.09). Associations were found in the moderate-to-high transmission setting (Table 6), children who visited on a weekday compared with the weekend were less likely to be referred (OR = 0.76; 95% CI = 0.65–0.89; *P* = 0.01), as were children who visited during the rainy season compared with the dry season (OR = 0.85; 95% CI = 0.73–0.99; *P* = 0.04). Children who visited within 24 hours of their symptom onset were also less likely to be referred (OR = 0.76; 95% CI = 0.61–0.94; *P* = 0.01) and referral was more likely when children slept under a net the previous night compared with children not sleeping under a net (OR = 1.41; 95% CI = 1.08–1.85; *P* = 0.011). In the low-transmission setting, the only factor in addition to ACT prescription independently associated with referral was distance. Children residing in villages further away from health centers were more likely to be referred (*P* = 0.001) compared with children living closer to health centers (Table 7).

## Discussion

This study demonstrates that CHWs trained to use mRDTs for malaria diagnosis refer children to health centers more frequently than CHWs using a presumptive clinical diagnosis. In both arms of the trials, CHWs were trained to refer based on the child's presenting signs and symptoms and thus referral rates were expected to be similar across the two arms; however, we found the mRDT result and prescription of an ACT affected the pattern of referral. In both transmission settings, almost all children who were diagnosed presumptively without an mRDT received an ACT and very few were referred. Similarly, children who were mRDT positive and treated with an ACT were rarely referred for other conditions. In contrast, referral was more likely in mRDT-negative children, who usually did not receive an ACT. These data suggest that although mRDT use can result in more referrals overall, the possibility of coinfections and other illnesses may be still overlooked in mRDT-positive children and the opportunity for early detection and referral is missed. Because the presence of referral signs and symptoms in children and their environment is unlikely to be affected by the method of diagnosis used by CHWs, this suggests that use of mRDTs encourages CHWs to consider alternative diagnoses if an ACT was not prescribed.

There were some observed differences between the two transmission settings. Referral was more frequent in the mRDT arm of the low-transmission setting compared with the mRDT arm of the moderate-to-high transmission setting. CHWs were trained to refer all children receiving prereferral treatment rectal artesunate; despite this, 14% of children receiving rectal artesunate in the moderate-to-high transmission setting were not referred. In contrast, almost all children receiving rectal artesunate in the low-transmission setting were referred. Poor adherence to the guidelines regarding the use of rectal artesunate in a community-based setting is a concern. Failure to refer children immediately to the nearest health center for further management when they are mRDT positive, have severe malaria signs and symptoms, and given rectal artesunate increases the child's risk of health complications, morbidity, and death.

The tendency of CHWs to report very few signs and symptoms on the referral form for children diagnosed presumptively or mRDT positive, compared with the greater range reported for children who were mRDT negative, may be partially explained by ACT prescription. Nearly all children in these two groups (presumptive and mRDT positive) received an ACT and CHWs may have considered the child as treated and they may, therefore, have been less likely to observe and record other signs and symptoms requiring referral. In contrast, when the child is mRDT negative and the CHW decides not to give the child an ACT, CHWs may attempt to identify more signs and symptoms of disease. It is also possible that children who were mRDT negative may have presented with a more complex and obvious set of clinical signs and symptoms compared with mRDT positive and presumptively diagnosed children who may have only presented with one or two discrete referral signs and symptoms. CHWs may increase the risk of further complications when ACTs are prescribed to children who are mRDT positive but fail to identify other clinical symptoms requiring referral. Unfortunately, in this study, we cannot know if CHWs were more likely to have missed signs and symptoms for referral in mRDT-positive children, because we did not directly observe CHWs practice of identifying referral signs and symptoms in these children. Nevertheless, the data presented here are suggestive of a possible concern, and further research, which includes observation of the clinical encounters at community level, is needed.

Although the factors independently associated with referral differed between the two transmission settings, prescription of an ACT was negatively associated with referral in both settings. Indeed, once ACTs were entered into multivariable model, differences according to the method of diagnosis (presumptive or mRDT) were reduced and no longer reached statistical significance. In the moderate-to-high transmission setting, other factors associated with a lower likelihood of referral included visits to CHWs during the weekend compared with a weekday, and during the rainy season compared with the dry season. These findings might be explained by perceived barriers to accessing health care in the area; during the weekend, health centers were often not open nor were they staffed with health workers and during the rainy season, roads and paths may become difficult to use to access the health centers. Given these barriers, CHWs may have been reluctant to refer. The multivariable analysis also found some evidence that children who presented within 24 hours of their symptoms starting were less likely to be referred. This could indicate that CHWs were waiting to see if the child's signs and symptoms worsened over the course of a day and only referred them after 24 hours if they worsened. Paradoxically, there was also some evidence to suggest that sleeping under a mosquito net the previous night increased the chance of being referred, the reasons for which are unclear. In the low-transmission setting, we also found the main driver of referral to be the prescribing pattern of ACTs and the only additional factor associated with referral was the distance of the village to the nearest health center. CHWs living in villages further away from public health centers were more likely to refer children than those living in villages closer to health centers. This observation might also have been affected by the presence of a privately run mission hospital that was located at the outskirts of the subcounty and further away from the more centrally located public health facilities in the low-transmission subcounty.

This and a number of other contextual factors differed between transmission settings, which may partly explain the tendency for CHWs in the low-transmission setting to refer more frequently than CHWs in the moderate-to-high transmission setting. First, the type of signs and symptoms that children presented with may differed between the two transmission settings. More children in the low-transmission setting were reported to have signs and symptoms of severe illness compared with children in the moderate-to-high transmission setting, which may have resulted in CHWs in the low-transmission setting referring more frequently than CHWs in the moderate-to-high transmission. The higher frequency of severe signs and symptoms in the low-transmission setting could reflect a lack of acquired immunity to malaria among young children living in this epidemic-prone area of the Ugandan highlands. Second, CHWs in the moderate-to-high transmission setting experienced a high number of visits and through experience they may have judged that they knew when it was safe not refer a child visit. In contrast, CHWs in the low-transmission setting experienced substantially fewer visits than CHWs in the moderate-to-high transmission setting, and may have felt less confident in their diagnostic competence and/or ability to make judgments about when it might be safe not to refer a child. Finally, in the low-transmission setting, CHWs were also able to refer patients to a large privately run mission hospital, which also coordinated a community-based health insurance scheme in the area. The scheme may have facilitated CHWs tendency to refer, being more confident knowing that health services and drugs would be available and that caretakers would comply with the referral advice.

When interpreting these findings, there are limitations that ought to be considered. CHWs often did not report the signs and symptoms for referral and very few referral forms were collected from CHWs compared with the treatment recording forms. CHWs reported child characteristics, fever history, treatment, and referral decisions (including whether it was a severe or nonsevere referral) on the treatment recording forms, whereas the referral forms captured the specific signs and symptoms for referral and were given to the caretaker to take to the health center. There is no obvious reason why very few referral forms were completed by CHWs, but some qualitative evidence from comments recorded by CHWs on the treatment forms suggests caretakers often refused referral forms and did not want to be referred to health centers. It is thus possible that CHWs only informed patients verbally that additional treatment from a health facility was advisable. Therefore, the data on referral signs and symptoms recorded on the forms may not be representative of all children who were referred by CHWs. Finally, in this analysis, we did not examine whether CHWs made the appropriate decision to refer. An analysis of referral in children with measured temperature ≥ 38.5°C (an eligibility criterion for severe referral) in this study suggests that CHW adherence to referral guidelines was poor and CHWs failed to refer children who were eligible for referral (Lal S, and others, 2016 *Malaria Journal* in press). Therefore, the combination of underreporting of referrals and failure to refer children who were eligible for referral may underestimate the true number of children who should have been referred. In addition to the data reporting practices by CHWs, there were also differences in the statistical analyses of each trial. In the moderate-to-high transmission setting, a modified intention to treat analysis was undertaken because after randomization, three of the 63 clusters (9/189 CHWs) withdrew from the study and did not provide data for the final analysis. Postrandomization withdrawal of clusters that were not included in the analysis may bias the results because CHWs in the clusters that withdrew from the study may have differed from those that did not withdraw. Therefore, some of the differences in referrals CHWs made between the two arms may not have been due to the intervention, but due to the differences between CHWs in each trial that remained in the study. However, in low-transmission setting trial, CHWs in all clusters contributed data to the primary outcome and an intention-to-treat analysis was used. Because of the different analytical approaches used, findings from the trial in the moderate-to-high transmission setting may not be directly comparable with the findings from the low-transmission trial. Although this study had limitations, similar patterns of referral and a strong relationship between prescription of ACT and referral were observed in both trials.

Despite the limitations, these results are consistent with previous studies that compared referral practices from CHWs trained to use either an mRDT or a presumptive diagnosis for malaria. A randomized crossover trial in Tanzania found CHWs trained to use mRDTs were more than four times more likely to refer children compared with CHWs trained to use a presumptive diagnosis.^11^ However, this previous study did not report whether the proportion of referrals differed by mRDT-positive or -negative results. Our results were also consistent with a study in Sierra Leone that found referral was more common among mRDT-negative patients compared with mRDT-positive children.^10^ Our study expands on these previous works by describing CHWs referral patterns over a larger time span, in two contrasting malaria-transmission settings and exploring other factors in addition to the diagnosis method that might affect the CHW's decision to refer.

These trials were designed and conducted before iCCM became national policy for many SSA countries and the training in this study related to malaria, thus all the findings may not be generalizable to iCCM programs, which also include the management of pneumonia and diarrhea in addition to malaria. However, there are a number of referral findings presented here that are of direct relevance to iCCM programs. First, routinely available data can be used to monitor and evaluate CHWs referral practices. Second, CHW training packages should emphasize the provision of referral advice to all children upon the identification of signs and symptoms requiring referral, and finally, training should strongly state that all children prescribed rectal artesunate should be referred for further management. Referral patterns and the reasons CHWs take to refer children should be examined in further studies along with the health outcomes of children referred.

## Conclusion

During the course of 2 years, we observed low referral rates in two contrasting malaria-transmission settings, but training CHWs to use mRDTs and ACTs increased the referral of children compared with CHWs trained to use a presumptive clinical diagnosis for malaria. Despite the increase, referral advice was not always given when rectal artesunate was prescribed as a prereferral treatment. We also found CHWs considered other factors alongside mRDTs and ACTs when considering to give referral advice. These findings suggest training on referral should be emphasized in iCCM programs being scaled-up in SSA and that additional research is required to examine whether CHWs referral decision-making is appropriate as well as the final health outcomes of referred children.

## Supplementary Material

Supplemental Datas.

## Figures and Tables

**Table 1:** Characteristics of children visiting CHWs

	Moderate-to-high transmission setting		Low-transmission setting	
	Control arm	Intervention arm	Control arm	Intervention arm
	Frequency (%)	Frequency (%)	Frequency (%)	Frequency (%)
Number of participating villages (clusters)	30	30	32	32
Number of CHWs	90	90	96	93
Total number of child visits to CHWs	10,599	7,501	2,404	1,097
Age group (years)				
< 1.0	2,042 (19.4)	1,528 (20.6)	587 (24.6)	297 (27.6)
1.0–2.9	3,982 (37.7)	3,113 (41.9)	970 (40.7)	478 (44.4)
3.0–4.9	4,506 (42.7)	2,751 (37.1)	817 (34.3)	295 (27.4)
5.0–15.0	21 (0.2)	33 (0.4)	11 (0.5)	6 (0.6)
Sex				
Male	5,329 (50.6)	3,907 (52.3)	1,264 (52.6)	550 (50.1)
Female	5,207 (49.4)	3,559 (47.7)	1,137 (47.3)	544 (49.6)
Slept under a net the previous night				
No	919 (8.7)	738 (9.8)	222 (9.2)	145 (13.2)
Yes	9,507 (89.7)	6,687 (89.1)	2,146 (89.3)	938 (85.5)
Resident in the same village as CHW				
No	785 (7.4)	690 (9.2)	311 (12.9)	191 (17.4)
Yes	9,751 (92.0)	6,792 (90.5)	2,085 (86.7)	901 (82.1)
Mean body temperature (°C)	37.43 (37.42–37.44)	37.38 (37.35–37.40)	37.42 (37.39–37.46)	37.21 (37.13–37.30)
Time of visit to CHW after onset of symptoms				
> 24 hours	586 (5.6)	1,089 (14.9)	271 (11.7)	232 (21.6)
Within 24 hours	9,799 (94.4)	6,222 (85.1)	2,050 (88.3)	844 (78.4)
Day of visit to a CHW				
Weekday	7,267 (68.6)	5,219 (69.6)	1,632 (67.9)	743 (67.7)
Weekend	3,332 (31.4)	2,282 (30.4)	772 (32.1)	354 (32.3)
Season of visit to a CHW				
Dry	4,630 (43.7)	2,761 (36.8)	969 (40.3)	378 (34.5)
Rainy	5,969 (56.3)	4,740 (63.2)	1,435 (59.7)	719 (65.5)
Village distance to nearest health facility (km)				
0.0–2.4	4,179 (39.8)	3,995 (53.7)	793 (34.6)	204 (19.8)
2.5–4.9	5,099 (48.6)	3,234 (43.5)	667 (29.1)	487 (47.3)
5.0–7.4	1,220 (11.6)	207 (2.8)	696 (30.3)	250 (24.3)
7.5–8.9	0 (0.0)	0 (0.0)	139 (6.1)	89 (8.6)

CHW = community health worker.

Data missing in the moderate-to-high-transmission setting for age 124 (48 control, 76 intervention), sex 98 (63 control, 35 intervention), slept under a net the previous night 249 (173 control, 76 intervention), resident in the same village as CHW 82 (63 control, 19 intervention), mean body temperature (°C) 267 (187 control, 80 intervention), and time of visit to CHW after onset of symptoms 404 (214 control, 190 intervention).

Data missing in the low-transmission setting for age 40 (19 control, 21 intervention), sex 6 (3 control, 3 intervention), slept under a net the previous night 50 (36 control, 14 intervention), resident in the same village as CHW 13 (8 control, 5 intervention), mean body temperature (°C) 26 (23 control, 3 intervention), and time of visit to CHW after onset of symptoms 104 (83 control, 21 intervention).

**Table 2:** Children referred in each arm, their mRDT test result, and ACT treatment received in the moderate-to-high transmission setting

	Total visits	No. referred (%)	No. of nonsevere referrals (%)*	No. of severe referrals (%)*
Trial arm				
Control (presumptive arm)	10,599	109 (1.0)	20 (24.7)	61 (75.3)
Intervention (mRDT arm)	7,501	2,651 (35.3)	1,562 (62.6)	932 (37.4)
Within intervention arm				
mRDT negative	4,147	2,558 (61.7)	1,536 (63.3)	892 (36.7)
mRDT positive	3,355	93 (2.8)	26 (39.4)	40 (60.6)
ACT prescription†				
ACT not prescribed	4,039	2,495 (61.8)	1,478 (62.7)	878 (37.3)
ACT prescribed	13,785	78 (0.6)	25 (53.2)	22 (46.8)
Rectal artesunate prescribed	70	60 (85.7)	5 (9.8)	46 (90.2)

ACT = artemisinin-based combination therapy; mRDT = malaria rapid diagnostic test.

* Percentage of type of referral (severe or nonsevere) of those who were referred, 185 visits missing type (severe or nonsevere) of referral.

† 206 missing ACT prescription.

**Table 3:** Children referred in each arm, their mRDT test result, and ACT treatment received in the low-transmission setting

	Total visits	No. referred (%)	No. of nonsevere referrals (%)*	No. of severe referrals (%)*
Trial arm				
Control (presumptive arm)	2,404	314 (13.1)	180 (67.7)	86 (32.3)
Intervention (mRDT arm)	1,097	782 (71.3)	444 (58.3)	317 (41.7)
Within intervention arm				
mRDT negative	1,064	770 (72.4)	439 (58.4)	313 (41.6)
mRDT positive	34	13 (38.2)	5 (50.0)	5 (50.0)
ACT prescription†				
ACT not prescribed	1,053	782 (74.3)	454 (59.8)	305 (40.2)
ACT prescribed	2,328	209 (9.0)	138 (79.8)	35 (20.2)
Rectal artesunate prescribed	59	58 (98.3)	12 (23.5)	39 (76.5)

ACT = artemisinin-based combination therapy; mRDT = malaria rapid diagnostic test.

* Percentage of type of referral (severe or nonsevere) of those who were referred, 69 visits missing type (severe or nonsevere) of referral.

† 61 missing ACT prescription.

**Table 4:** Nonsevere referral signs and symptoms of children visiting CHWs

	Moderate-to-high transmission setting		Low-transmission setting	
	Control arm	Intervention arm	Control arm	Intervention arm
	Frequency (%)	Frequency (%)	Frequency (%)	Frequency (%)
Nonsevere signs and symptoms for referral				
Fever in babies less than 4 months old	0 (0.0)	0 (0.0)	0 (0.0)	0 (0.0)
Fever that has lasted more than 7 days	0 (0.0)	16 (4.8)	1 (6.7)	3 (3.1)
Fever with measured temperature of > 37°C and mRDT negative	0 (0.0)	214 (64.3)	0 (0.0)	37 (38.1)
Vomiting and diarrhea	1 (100.0)	30 (9.0)	7 (46.7)	13 (13.4)
Blood in feces or urine	0 (0.0)	2 (0.6)	0 (0.0)	0 (0.0)
Pain when passing urine, or frequent urination	0 (0.0)	7 (2.1)	1 (6.7)	3 (3.1)
Wounds or burns	0 (0.0)	8 (2.4)	0 (0.0)	2 (2.1)
Skin abscess	0 (0.0)	3 (0.9)	0 (0.0)	0 (0.0)
Painful swellings or lumps in the skin	0 (0.0)	3 (0.9)	1 (6.7)	1 (1.0)
Ear infection (runny ear or child pulling at ear)	0 (0.0)	1 (0.3)	0 (0.0)	0 (0.0)
Sticky or red eyes	0 (0.0)	15 (4.5)	0 (0.0)	1 (1.0)
Other nonsevere signs and symptoms*	0 (0.0)	34 (10.2)	5 (33.3)	37 (38.1)
Total number of nonsevere signs and symptoms reported	1	333	15	97
Total number of nonsevere referrals forms	1	108	16	24
Mean number of signs and symptoms reported per nonsevere referral form	1.0	3.1	0.9	4.0

CHW = community health worker.

* Other nonsevere signs and symptoms in the moderate-to-high transmission setting intervention arm included cough and flu (14), difficulty in breathing (1), swollen legs and eyes (1), headache (1), worms (1), high temperature (17), and no fever (1); other nonsevere signs and symptoms in the low-transmission setting intervention arm included cough and flu (11), difficulty in breathing (1), high temperature (19), unable to breast feed (1), mouth wounds (1), control arm, and cough (5).

**Table 5:** Severe referral signs and symptoms of children visiting CHWs

	Moderate-to-high transmission setting		Low-transmission setting	
	Control arm	Intervention arm	Control arm	Intervention arm
	Frequency (%)	Frequency (%)	Frequency (%)	Frequency (%)
Severe signs and symptoms for referral				
Illness in child below 2 months	0 (0.0)	2 (0.6)	0 (0.0)	1 (1.7)
Convulsions or fits now or within the past 2 days	0 (0.0)	21 (5.9)	0 (0.0)	2 (3.4)
Coma/loss of consciousness	0 (0.0)	6 (1.7)	1 (3.0)	0 (0.0)
Patient is confused or very sleepy—cannot be woken	1 (16.7)	4 (1.1)	1 (3.0)	1 (1.7)
Extreme weakness unable to stand or sit without support	1 (16.7)	10 (2.8)	1 (3.0)	2 (3.4)
Very hot with temperature of 38.5°C or more	2 (33.3)	78 (22.0)	13 (39.4)	18 (30.5)
Very cold with temperature of 35.0°C or less	0 (0.0)	10 (2.8)	1 (3.0)	2 (3.4)
Vomiting everything—cannot keep down food or drink	0 (0.0)	36 (10.2)	3 (9.1)	5 (8.5)
Not able to drink or breastfeed	0 (0.0)	52 (14.7)	1 (3.0)	7 (11.9)
Severe anemia very pale palms, fingernails, eyelids	0 (0.0)	18 (5.1)	1 (3.0)	1 (1.7)
Yellow eyes	0 (0.0)	9 (2.5)	1 (3.0)	0 (0.0)
Difficulty in breathing	2 (33.3)	69 (19.5)	8 (24.2)	14 (23.7)
Severe dehydration	0 (0.0)	7 (2.0)	0 (0.0)	1 (1.7)
Other severe signs and symptoms‡	0 (0.0)	32 (9.0)	2 (6.1)	5 (8.5)
Total number of severe signs and symptoms reported	6	354	33	59
Total number of severe referral forms	2	149	16	24
Mean number of signs and symptoms reported per severe referral form	3.0	2.4	2.1	2.5
Total number of severe and nonsevere signs and symptoms reported	7	687	48	156
Total nonsevere and severe referral forms^*^	3	257	31	78
Total referrals made	109	2,651	314	782

CHW = community health worker.

‡ Other severe signs and symptoms included in the moderate-to-high transmission setting intervention arm: cough and flu (19), diarrhea (4), dysentery (2), burns (1), eye problems (2), painful ear (3), eating problem (1), yellow skin (1), and vomiting (1); other severe signs and symptoms included in the low-transmission setting intervention arm: abdominal pain (1), constipation (1), cough (1), difficulty in breathing (1), eye problems (1), control arm, abdominal pain (1), and eye problems (1).

**Table 6:** ORs for referral of children in the moderate-to-high transmission setting

Variables	Total visits	Referrals made (%)	Unadjusted OR (95% CI)	*P* value	Adjusted OR (95% CI)	*P* value
Study arm						
Control	10,599	109 (1.0)	1		1	
mRDT intervention	7,501	2,651 (35.3)	61.90 (38.40–99.77)	< 0.001	2.07 (0.97–4.41)	0.060
Age group (years)						
< 1.0	3,570	804 (22.5)	1		1	
1.0–2.9	7,095	1,148 (16.2)	0.55 (0.48–0.62)	< 0.001	0.88 (0.73–1.05)	
3.0–4.9	7,257	771 (10.6)	0.31 (0.27–0.36)		0.79 (0.65–0.97)	0.140
5.0–15.0	54	10 (18.5)	0.43 (0.19–0.95)		0.68 (0.18–2.53)	
Sex						
Male	9,236	1,430 (15.5)	1		1	
Female	8,766	1,309 (14.9)	0.93 (0.84–1.02)	0.133	0.95 (0.82–1.11)	0.528
Slept under a net the previous night						
No	1,657	263 (15.9)	1		1	
Yes	16,194	2,450 (15.1)	1.52 (1.27–1.83)	< 0.001	1.41 (1.08–1.85)	0.011
Resident in the same village as CHW						
No	1,475	310 (21.0)	1		1	
Yes	16,543	2,442 (14.8)	1.03 (0.87–1.22)	0.751	0.91 (0.69–1.21)	0.531
Time of visit to CHW after onset of symptoms						
> 24 hours	1,675	399 (23.8)	1		1	
Within 24 hours	16,021	2,281 (14.2)	0.80 (0.69–0.92)	0.002	0.76 (0.61–0.94)	0.011
ACT prescription						
No ACT	4,039	2,495 (61.8)	1		1	
ACT	13,785	78 (0.6)	0.00 (0.00–0.00)	< 0.001	0.00 (0.00–0.00)	< 0.001
Day of visit to a CHW						
Weekday	12,486	1,977 (15.8)	1		1	
Weekend	5,614	783 (13.9)	0.86 (0.77–0.95)	0.005	0.76 (0.65–0.89)	0.001
Season of visit to a CHW						
Dry	7,391	1,110 (15.0)	1		1	
Rainy	10,709	1,650 (15.4)	0.79 (0.71–0.87)	< 0.001	0.85 (0.73–0.99)	0.042
Village distance to nearest health center (km)						
0.0–2.4	8,174	1,548 (18.9)	1		1	
2.5–4.9	8,333	1,116 (13.4)	1.15 (0.84–1.59)		1.28 (0.81–2.03)	
5.0–7.4	1,427	72 (5.0)	0.97 (0.37–2.52)	0.647	1.24 (0.44–3.49)	0.579
7.5–8.9	NA	NA	NA		NA	

ACT = artemisinin-based combination therapy; CHW = community health worker; CI = confidence interval; mRDT = malaria rapid diagnostic test; OR = odds ratio.

**Table 7:** ORs for referral of children in the low-transmission setting

Variables	Total visits	Referrals made (%)	OR (95% CI)	*P* value	Adjusted OR (95% CI)	*P* value
Study arm						
Control	2,404	314 (13.1)	1		1	
mRDT intervention	1,097	782 (71.3)	28.45 (15.41–52.49)	< 0.001	2.07 (0.90–4.79)	0.090
Age group (years)						
< 1.0	884	313 (35.4)	1		1	
1.0–2.9	1,448	467 (32.3)	0.75 (0.59–0.95)	0.002	0.90 (0.68–1.20)	
3.0–4.9	1,112	291 (26.2)	0.64 (0.49–0.83)		0.74 (0.54–1.01)	0.285
5.0–15.0	17	9 (52.9)	2.23 (0.61–8.15)		0.74 (0.13–4.32)	
Sex						
Male	1,814	564 (31.1)	1		1	
Female	1,681	527 (31.4)	0.93 (0.77–1.13)	0.463	0.94 (0.74–1.18)	0.579
Slept under a net the previous night						
No	367	124 (33.8)	1		1	
Yes	3,084	951 (30.8)	1.04 (0.76–1.43)	0.788	1.12 (0.77–1.64)	0.552
Resident in the same village as CHW						
No	502	177 (35.3)	1		1	
Yes	2,986	916 (30.7)	1.11 (0.85–1.47)	0.440	1.19 (0.81–1.73)	0.372
Time of presentation to CHW after onset of symptoms						
> 24 hours	503	212 (42.1)	1		1	
Within 24 hours	2,894	851 (29.4)	0.83 (0.63–1.09)	0.180	0.89 (0.64–1.22)	0.464
ACT prescription						
No ACT	1,053	782 (74.3)	1		1	
ACT	2,328	209 (9.0)	0.02 (0.01–0.03)	< 0.001	0.03 (0.02–0.05)	< 0.001
Day of visit to a CHW						
Weekday	2,375	763 (32.1)	1		1	
Weekend	1,126	333 (29.6)	0.82 (0.67–1.01)	0.058	0.88 (0.69–1.13)	0.321
Season of visit to a CHW						
Dry	1,347	403 (29.9)	1		1	
Rainy	2,154	693 (32.2)	1.01 (0.82–1.23)	0.960	0.98 (0.77–1.25)	0.859
Village distance to nearest health center (km)						
0.0–2.4	997	285 (28.6)	1		1	
2.5–4.9	1,154	384 (33.3)	0.75 (0.34–1.66)		0.42 (0.21–0.83)	
5.0–7.4	946	236 (24.9)	1.36 (0.52–3.58)	0.111	0.98 (0.46–2.12)	0.001
7.5–8.9	228	124 (54.4)	3.66 (0.88–15.19)		3.23 (1.01–10.38)	

ACT = artemisinin-based combination therapy; CHW = community health worker; CI = confidence interval; mRDT = malaria rapid diagnostic test; OR = odds ratio.
