# Supplementary material for: Referral Patterns of Community Health Workers Diagnosing and Treating Malaria: Cluster-Randomized Trials in Two Areas of High- and Low-Malaria Transmission in Southwestern Uganda
Source: Am J Trop Med Hyg. 2016 Dec 7;95(6):1398–408. doi: 10.4269/ajtmh.16-0598 (PMC5154457; doi:10.4269/ajtmh.16-0598)
Supplement: Supplementary file 1 [file SD4.pdf]

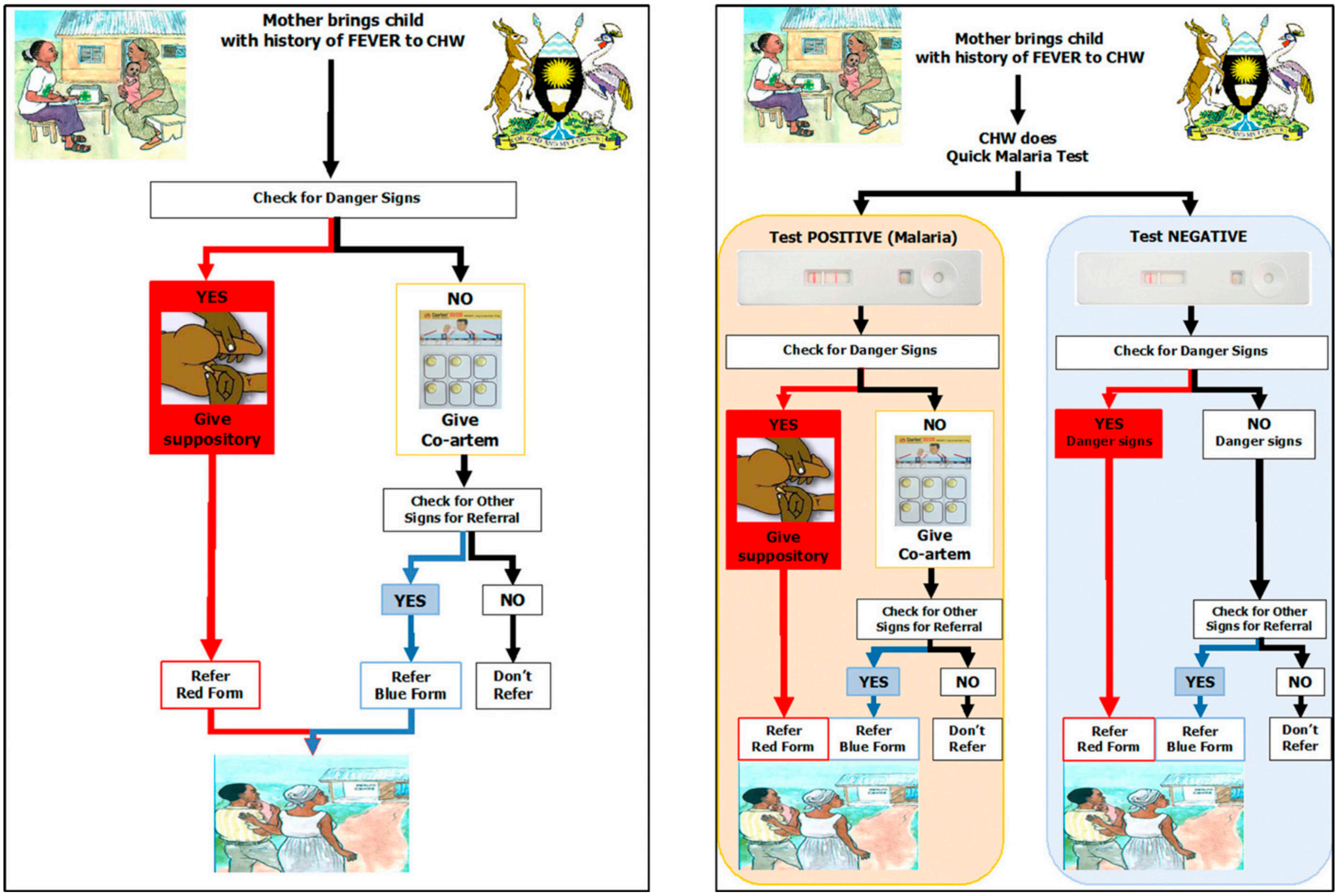

Supplemental Figure 1. Pictorial treatment algorithm for community health workers (CHWs) in (**A**) malaria rapid diagnostic test intervention arm and (**B**) control arm.

Supplemental Table 1

Referral signs and symptoms of children visiting CHWs by mRDT results, in the moderate-to-high transmission setting

	Moderate-to-high transmission setting*		
	Intervention arm	mRDT negative	mRDT positive
	Frequency (%)	Frequency (%)	Frequency (%)
Nonsevere signs and symptoms for referral			
Fever in babies less than 4 months old	0 (0.0)	0 (0.0)	0 (0.0)
Fever that has lasted more than 7 days	16 (4.8)	16 (4.9)	0 (0.0)
Fever with measured temperature of > 37°C and mRDT negative	214 (64.3)	214 (65.0)	0 (0.0)
Vomiting and diarrhea	30 (9.0)	29 (8.8)	1 (25.0)
Blood in feces or urine	2 (0.6)	2 (0.6)	0 (0.0)
Pain when passing urine, or frequent urination	7 (2.1)	7 (2.1)	0 (0.0)
Wounds or burns	8 (2.4)	8 (2.4)	0 (0.0)
Skin abscess	3 (0.9)	3 (0.9)	0 (0.0)
Painful swellings or lumps in the skin	3 (0.9)	3 (0.9)	0 (0.0)
Ear infection (runny ear or child pulling at ear)	1 (0.3)	1 (0.3)	0 (0.0)
Sticky or red eyes	15 (4.5)	13 (4.0)	2 (50.0)
Other nonsevere signs and symptoms†	34 (10.2)	33 (10.0)	1 (25.0)
Total number of nonsevere signs and symptoms reported	333	329	4
Total number of nonsevere referrals forms	108	103	5
Mean number of signs and symptoms reported per referral form	3.1	3.2	0.8
Severe signs and symptoms for referral			
Illness in child below 2 months	2 (0.6)	2 (0.6)	0 (0.0)
Convulsions or fits now or within the past 2 days	21 (5.9)	19 (5.7)	2 (10.0)
Coma/loss of consciousness	6 (1.7)	5 (1.5)	1 (5.0)
Patient is confused or very sleepy—cannot be woken	4 (1.1)	4 (1.2)	0 (0.0)
Extreme weakness unable to stand or sit without support	10 (2.8)	9 (2.7)	1 (5.0)
Very hot with temperature of 38.5°C or more	78 (22.0)	73 (21.9)	5 (25.0)
Very cold with temperature of 35.0°C or less	10 (2.8)	10 (3.0)	0 (0.0)
Vomiting everything—cannot keep down food or drink	36 (10.2)	35 (10.5)	1 (5.0)
Not able to drink or breastfeed	52 (14.7)	48 (14.4)	4 (20.0)
Severe anemia very pale palms, fingernails, eyelids	18 (5.1)	17 (5.1)	1 (5.0)
Yellow eyes	9 (2.5)	8 (2.4)	1 (5.0)
Difficulty in breathing	69 (19.5)	65 (19.5)	4 (20.0)
Severe dehydration	7 (2.0)	7 (2.1)	0 (0.0)
Other severe symptoms‡	32 (9.0)	32 (9.6)	0 (0.0)
Total number of signs and symptoms reported	354	334	20
Total number of severe referral forms	149	142	7
Mean number of symptoms reported per severe referral form	2.4	2.4	2.9
Total number of severe and nonsevere symptoms reported	687	663	24
Total nonsevere and severe referral forms	257	245	12
Total referrals made	2,651	2,558	93

CHW = community health worker; mRDT = malaria rapid diagnostic test.

*Percentages are calculated based on the total number of signs and symptoms reported.

†Other nonsevere signs and symptoms in children mRDT negative included cough and flu (14), difficulty in breathing (1), swollen legs and eyes (1), headache (1), worms (1), and high temperature (15); other nonsevere symptoms in children mRDT positive included cough and flu (1).

‡Other severe signs and symptoms included in children mRDT negative included cough and flu (17), diarrhea (4), dysentery (2), burns (1), eye problems (2), painful ear (3), eating problem (1), yellow skin (1), and vomiting (1).

Supplemental Table 2

Referral signs and symptoms of children visiting CHWs by mRDT results in the low-transmission setting

	Low-transmission setting*		
	Intervention arm	mRDT negative	mRDT positive
	Frequency (%)	Frequency (%)	Frequency (%)
Nonsevere signs and symptoms for referral			
Fever in babies less than 4 months old	0 (0.0)	0 (0.0)	0 (0.0)
Fever that has lasted more than 7 days	3 (3.1)	3 (3.2)	0 (0.0)
Fever with measured temperature of > 37°C and mRDT negative	37 (38.1)	37 (38.9)	0 (0.0)
Vomiting and diarrhea	13 (13.4)	13 (13.7)	0 (0.0)
Blood in feces or urine	0 (0.0)	0 (0.0)	0 (0.0)
Pain when passing urine, or frequent urination	3 (3.1)	3 (3.2)	0 (0.0)
Wounds or burns	2 (2.1)	2 (2.1)	0 (0.0)
Skin abscess	0 (0.0)	0 (0.0)	0 (0.0)
Painful swellings or lumps in the skin	1 (1.0)	1 (1.1)	0 (0.0)
Ear infection (runny ear or child pulling at ear)	0 (0.0)	0 (0.0)	0 (0.0)
Sticky or red eyes	1 (1.0)	1 (1.1)	0 (0.0)
Other nonsevere signs symptoms†	37 (38.1)	35 (36.8)	2 (100.0)
Total number of nonsevere signs and symptoms reported	97	95	2
Total number of nonsevere referrals forms	54	52	2
Mean number of signs and symptoms reported per referral form	1.8	1.8	1.0
Severe signs and symptoms for referral			
Illness in child below 2 months	1 (1.7)	1 (1.8)	0 (0.0)
Convulsions or fits now or within the past 2 days	2 (3.4)	2 (3.5)	0 (0.0)
Coma/loss of consciousness	0 (0.0)	0 (0.0)	0 (0.0)
Patient is confused or very sleepy—cannot be woken	1 (1.7)	1 (1.8)	0 (0.0)
Extreme weakness unable to stand or sit without support	2 (3.4)	2 (3.5)	0 (0.0)
Very hot with temperature of 38.5 or more	18 (30.5)	17 (29.8)	1 (50.0)
Very cold with temperature of 35.0 or less	2 (3.4)	2 (3.5)	0 (0.0)
Vomiting everything—cannot keep down food or drink	5 (8.5)	5 (8.8)	0 (0.0)
Not able to drink or breastfeed	7 (11.9)	7 (12.3)	0 (0.0)
Severe anemia very pale palms, fingernails, eyelids	1 (1.7)	1 (1.8)	0 (0.0)
Yellow eyes	0 (0.0)	0 (0.0)	0 (0.0)
Difficulty in breathing	14 (23.7)	13 (22.8)	1 (50.0)
Severe dehydration	1 (1.7)	1 (1.8)	0 (0.0)
Other severe signs and symptoms‡	5 (8.5)	5 (8.8)	0 (0.0)
Total number of severe signs and symptoms reported	59	57	2
Total number of severe referral forms	24	23	1
Mean number of signs and symptoms reported per severe referral form	2.5	2.5	2.0
Total number of severe and nonsevere signs and symptoms reported	156	152	4
Total nonsevere and severe referral forms	78	75	3
Total referrals made	782	769	13

*Percentages are calculated based on the total number of signs and symptoms reported.

†Other nonsevere signs and symptoms of mRDT-negative children included cough and flu (8), vomiting (1), high temperature (20), difficulty in breathing (2), eating problem (1), headache (2), and worms (1); other non-evere signs and symptoms of mRDT-positive children included cough and flu (1) and headache (1).

‡Other severe signs and symptoms of children in the intervention arm included abdominal pain (1), constipation (1), cough (1), difficulty in breathing (1), and eye problems (1).
